# New products

**DOI:** 10.1155/S1463924685000244

**Published:** 1985


Journal of Automatic Chemistry/*Journal of Clinical Laboratory Automation*, Vol. 7, No. 2 (Apr–Jun 1985), pp. 104–119

### New products

### Laboratory Robotics Club

The Laboratory Robotics Club was formed last year by representatives from the Laboratory of the Government Chemist, industrial laboratories, universities and polytechnics. The club will sponsor R & D studies on the use of small robots in laboratories and will also support the exchange of information about the application of robotics in a lab. environment. The research and development activities that will be supported by the Club are of two types:

Short-term projects, mainly applications-orientated, that are designed to make maximum use of existing technology.

Medium- or long-term projects aimed at developing novel technology, both hardware and software. Ultimately such work will reach a state where it is suitable for commercial exploitation.

Other Club activities will include:

Comparative evaluations of robotics hardware, control software and applications techniques.

Setting up of standards related to the activities of the Club.

The organization of courses in robotics technology and applications (for which fees may be charged to cover running costs).

A regular newsletter.

Participation in an information interchange scheme, including the production of occasional state-of-the-art reports.

Visits and meetings.

The maintenance of a register of consultants in robotics technology and applications, the cost of these services being a matter for negotiation between the member and the consultant.

The Club's activities are financed by the subscriptions of members who are drawn from industrial bodies and public institutions. The Department of Trade and Industry also provides financial support for the R & D objectives of the Club.

The Club has a single grade of membership. Members are subscribing companies or organizations who each have a single vote. Members pay a fee of £400 per annum. Members joining within three years of the Club's formation will pay an entry fee equivalent to the amount that they would have paid in subscriptions had they been members of the Club *ab initio*, i.e. members joining in the second and third years of the Club's life will pay entrance fees of £400 and £800 respectively. This will entitle them to access to all previously published Club publications and R & D reports. New members joining the Club after the passage of three years will pay an entrance fee at a rate to be determined by the Management Committee.

*Application forms from the Laboratory of the Government Chemist, Cornwall House, Stamford Street, London SE1 9NQ. Tel.: 01 928 7900.*

**Circle No. 21 on Reader Enquiry Card**

### Graphics and kinetics software

Perkin-Elmer's Lambda 7 spectrophotometer has two new software packages: graphics and kinetics. The Lambda 7's high-resolution monitor provides good representation of graphics data in both scanning and fixed wavelength modes. With the graphics software, spectra can be stored in up to six different memory files, each of which can be addressed independently for automatic viewing on screen, or copying on to a high-speed printer/plotter. Spectra can be added or subtracted, and multiplication factors applied for comparison of data. Derivative spectra from stored absorbance or transmittance data can also be calculated. A cursor is used to display wavelength and photometric values, and small portions of a spectrum can be expanded to full scale with a zoom feature.

Using the enzyme kinetics facility, lag times, number of data points and intervals between data points (which can be as small as 0·01 min) can all be selected. An enzyme factor allows units of enzyme activity to be calculated. In addition, the software calculates the delta absorbance value. Standard deviation of the data points from a straight line is included, and a linearity factor, based on the slope of the reaction, aids validation of results.

Both graphics and kinetics software are compatible with the six cell programmer of the instrument for automated analysis. Data from each cell can be output to the monitor and printer/plotter.

An update kit is available for existing Lambda 7s.

*For further information contact Steve Upstone, Perkin-Elmer Ltd, Post Office Lane, Beaconsfield, Buckinghamshire HP9 1QA, UK. Tel.: 04946 6161.*

**Circle No. 22 on Reader Enquiry Card**

### Latest GC application news bulletins

Packard Instrument Ltd offers its two most recent *GC Application News Bulletins* free of charge. Each bulletin describes a particular analysis; all relevant experimental conditions are given and the resultant chromatogram is illustrated. The first describes the characterization of beeswax using the capillary liquid on-column injection technique and a deactivated fused silica retention gap with single ferrule column connection. The second describes the analysis of various trade preparations of pyrethroid insecticides using the capillary liquid on-column injection technique and an electron capture detector.

*Copies of either or both of the Bulletins can be obtained from Packard Instrument Limited, 13/17 Church Road, Caversham, Reading, Berkshire RG4 7AA, UK.*

**Circle No. 23 on Reader Enquiry Card**

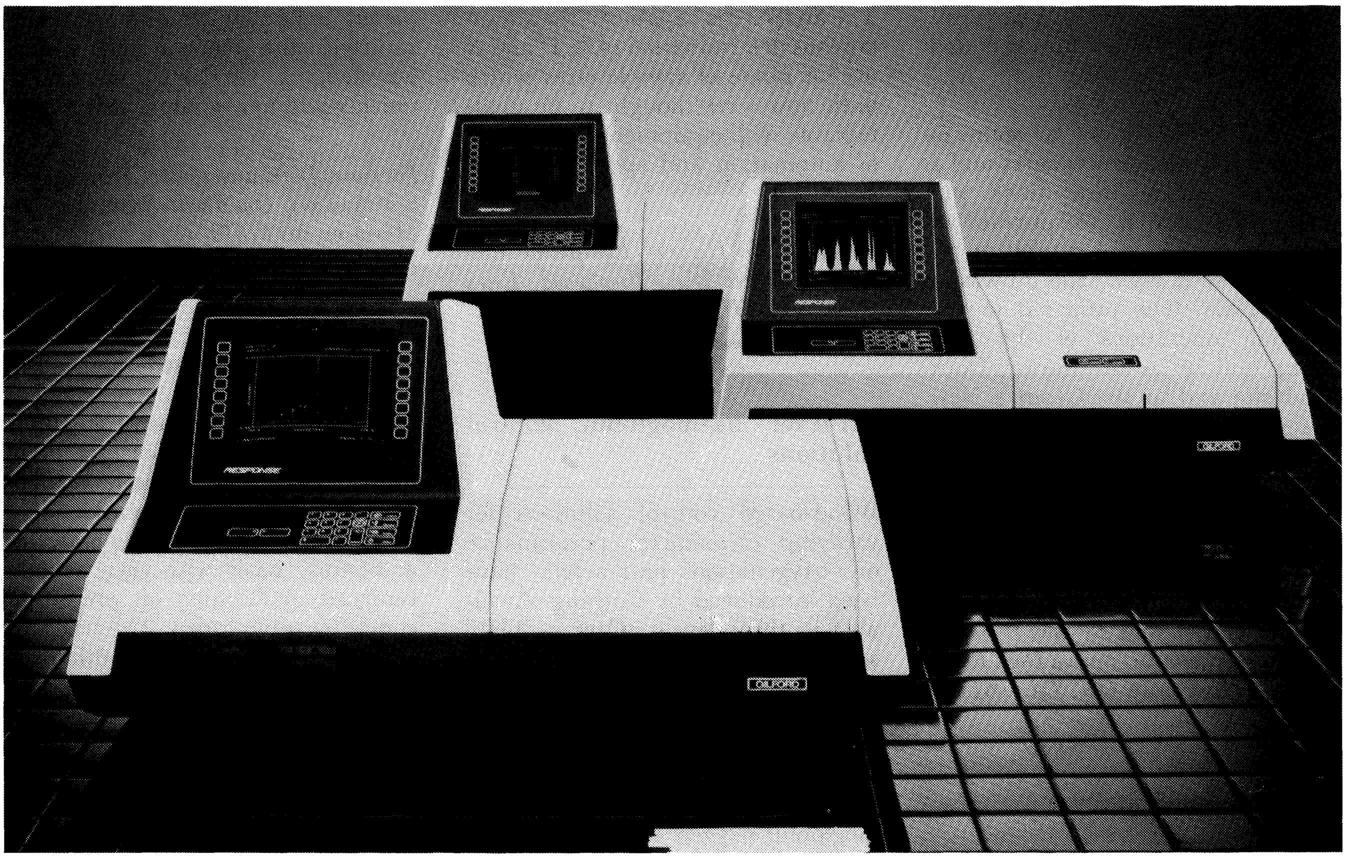

*The Corning Gilford Response range of UV/Vis spectrophotometers has now been expanded to encompass three separate models. Common to all models is an autoranging photometer: transverse sample compartment; high-resolution graphics; data storage facilities and a 16-bit resident computer. The basic Response is supplied complete with a single cell holder and programs for: wavelength scanning; kinetics; time scanning; multi-wavelength analysis; standard curves and sample read. The second unit has an automatic six-position cell holder and has the capability of adding gel scanning, rapid sampling and a six-position thermoset cuvette holder for electronic control of sample temperature. The most advanced Response has two disk drives for achival date storage and recall, together with extended software applications. Details from Corning Ltd, Halstead, Essex CO9 2DX, UK. Tel.: 0787 472461.*

**Circle No. 24 on Reader Enquiry Card**

### Chemputer 10

A low-cost computing-integrator system for use in gas and liquid chromatography has been introduced by Quadrant Scientific.

Based on the Apricot micro, the system works in conjunction with any GC or LC detector through a specially-designed plug-in-and-run interface module. The dedicated software package, written by Quadrant Scientific, provides user-friendly operating techniques; the system can be handled by staff without previous computer experience.

By matching integrator operation with a standard computer like the Apricot in this way, the company is offering a system with the control and flexibility that an integrator alone could not provide.

The software performs a wide range of functions allowing both area and height to be reported, as % area/height, together with normalization, internal or external standard, with linear or non-linear multi-level calibration, calibration/analysis sequencing and bracketing. Screen chromatograms are displayed using a 'split-screen' format which shows the whole run, plus current peaks at whatever size may be required. Storage of both raw and bunched data allows either true reintegration with different parameters or simply base-line reallocation. The system also incorporates a help facility.

The complete system consists of an Apricot computer with a high-resolution monitor, the laboratory software package and interface module, and a printer for hard-copy recording. Also covered by the highly competitive basic price is a range of general-purpose business software for the Apricot, including word-processing and spread-sheet analysis.

*Further information from Quadrant Scientific, 38 Brunswick Road, Gloucester GL1 1JJ, UK. Tel.: 0452 20306.*

**Circle No. 25 on Reader Enquiry Card**

### Kratos Analytical Instruments

Dr John Waldron and Mr Roger Speare, two directors of Kratos Limited, the Manchester-based analytical instrument manufacturers, and American co-director Malcolm Kahn, have led a management buy-out of the company from the US parent company – Kratos, Inc. of La Jolla, California, for $10M. A new company, Spectros International PLC, has been formed to acquire the mass spectrometry, surface science and liquid chromatography business of the Kratos Group —an acknowledged leader in analytical instrument technology operating in a world-wide market estimated to be worth over £25M.

The deal, funded by a team of directors and senior managers and a group of financial institutions, will enable the Group to pursue an expansion policy in a market sector which has seen rapid sales growth in recent years. The principal manufacturing operations of the new Group will be at the Barton Dock Road site and at the Group's New Jersey factory. Sales, currently in excess of £20M per year, are made in over 30 countries and the Group has sales and service organizations in its main markets – the US, the UK, FR Germany and France.

*More information from Dr John Waldron, Spectros International, Barton Dock Road, Urmston, Manchester, UK. Tel.: 061 865 4466.*

**Circle No. 26 on Reader Enquiry Card**

### CHN elemental analyser

The microprocessor-controlled Heraeus CHN elemental analyser has been further developed. The instrument permits a wide range of sample weights – as high as 200 mg. Rapid analysis, taking as little as 10 min, is aided by the incorporation of a high degree of automation, including automatic sample loading. The automatic magazine allows up to 49 samples to be pre-loaded. A dedicated microcomputer and interface, together with a specially developed standard program, allows the unit to be operated overnight unattended, providing a print-out of sample details, including sample numbers, weights, measured values and percentage composition.

The analyser has obvious applications in the microanalysis of organic compounds. In addition, the wide range of sample sizes makes it an ideal instrument for the examination of diverse materials, from plastics and pharmaceutical products to fuels and foodstuffs. The unit offers a lower detection limit of $10^{-3}$ mg absolute for nitrogen, $5 \times 10^{-4}$ mg for carbon and $10^{-4}$ mg for hydrogen. It makes possible the quantitative determination of any substance which is combustible in an oxygen stream at up to 1050°C. The analyser is capable of the simultaneous determination of C, H and N over a range of sample sizes which until now were thought to be unattainable with equipment of this level of automation and speed of operation.

*Details from Heraeus Equipment Ltd, Unit 9, Wates Way, Brentwood, Essex CM15 9TB, UK. Tel.: 0277 231511.*

**Circle No. 27 on Reader Enquiry Card**

### Tri-level haemoglobin control solutions

Blood-based control solutions for verifying co-oximeter performance on oxygenation parameters have been introduced by Corning. Available in three levels, 'Certain THb' solutions simulate values typical of anaemia, normal and polycythemic samples. Each 30-ampoule package contains an assay value card listing expected values for: total haemoglobin; carboxyhaemoglobin; methemoglobin; oxygen content and oxygen saturation. Supplied as a stabilised solution of haemoglobin, Certain THb requires no special storage or preparation; it can be used at room temperature with either the Corning 2500 or IL282 co-oximeters.

*More information from Corning Medical and Scientific, Corning Ltd, Halstead, Essex CO9 2DX, UK. Tel.: 0787 472461.*

**Circle No. 28 on Reader Enquiry Card**

### Allied software

Allied Analytical Systems have developed a software system, Multiquant, for the Plasma-200 ICP emission spectrometer. With the system up to 29 elements in an unknown sample can be determined within 5 min; up to 70 elements are possible with program modification. An operator can quickly calibrate Multiquant using a single standard solution which contains only three elements. For analyte elements not present in the standard, the computer calibrates by reference to one of the three elements using a predetermined ratio. Multiquant also features automatic spectral scans at each analytical wavelength (which reinforce the validity of analytical results for completely unknown samples), and versatility in the selection of analytical wavelengths (to help the analyst quickly add new analyte elements).

*For more information contact Allied Analytical Systems, One Burtt Road, Andover, Massachusetts 01810, USA.*

**Circle No. 29 on Reader Enquiry Card**

### National QC scheme

A national quality-control scheme is now available from Corning to all users of Confirm, a three-level blood gas analyser control solution. The scheme, 'Confirmation', operates on a monthly basis with results being returned to Corning on preprinted computer input forms. The results of the scheme are returned within three weeks and consist of single-page reports displaying graphically both precision and accuracy data. By using these reports performance can be reviewed at a glance for all instruments, all like instruments for the last month, lot to date and previous 12 months. Membership of Confirmation is currently free to all users ordering a 12-month supply of Confirm.

*More information from Corning (above).*

**Circle No. 30 on Reader Enquiry Card**

### Plasma hydride device

A continuous hydride generator, the PHD, which serves as an accessory to the Plasma 200 ICP Emission Spectrometer has been announced by Allied Analytical Systems. The PHD provides the following capabilities and features:

- Improves sensitivities and detection limits for hydride-forming elements, including arsenic, bismuth, germanium, antimony, selenium, tellerium and others.
- Permanent installation.
- Fast, simple switch-over between hydride generation and conventional nubulization; no mechanical disassembly and no need to extinguish the plasma discharge.
- Much less expensive than competitive hydride systems.

The PHD consists of a mixing chamber, a modified nebulizer, and a redesigned drain trap for the spray chamber. During hydride generation, pre-acidified sample and sodium borohydride reducing agent are drawn continuously through the two channels of the standard peristaltic pump, reacted together in the mixing chamber, and carried to the nebulizer/spray chamber. At the nebulizer, volatile hydride gases are mixed with the nebulizer propellant gas and carried to the plasma discharge, while the rest of the sample is nebulized normally.

*For more information, contact Allied Analytical Systems, One Burtt Road, Andover, Massachusetts 01810, USA. Tel.: 617 890 4300.*

**Circle No. 31 on Reader Enquiry Card**

### Spectrophotometer

Diode array spectrophotometers have always provided the fastest scan speeds, but at the expense of optical performance. Perkin-Elmer's new diode array spectrophotometer, the Lambda Array 3840, offers improved diode array technology for faster analysis and sets a new standard of optical performance. The 3840 scans and displays spectra from its 190–900 nm wavelength range within 2 s and provides spectral resolution of 0·25 nm. Stray light values of less than 0·05% ensure excellent photometric accuracy at high absorbance values. A choice of two scan modes allows the analyst to optimize speed and performance for all applications. In Survey Mode, resolution is fixed at 1·5 nm and a full range spectrum can be scanned and displayed on the colour monitor in less than 2 s; the wide scanning range is achieved through the use of deuterium and tungsten sources. In High Performance Mode, spectral resolution can be set at 0·25, 0·5, 1·0, 2·0, 4·0, or 8·0nm, and stray light is typically less than 0·02%. A 100 nm range spectrum can be displayed in under 2 s; in this mode, spectral bands separated by as little as 0·5 nm can be resolved easily and measured accurately.

Diode array spectrophotometers generally require a cell of standard path-length to be used—deviation from this causes recording errors. The optical system of the Lambda Array 3840 is unique in that it compensates for changes in path-length. Therefore, a wide range of path length cells, from 1 to 100mm, can be used, allowing precise analysis of difficult samples.

The Lambda Array 3840 is controlled by a Perkin-Elmer Series 7000 Professional Computer, equipped with colour monitor, dual floppy disk drives and a 10 megabyte Winchester hard disk. Interaction via assigned function keys and 'soft' keys located on the bezel of the colour monitor keeps operation fast and simple. All parameters are visible at a glance on the screen, so selection of operating conditions is made easy.

Perkin-Elmer's library of advanced UV/Vis application software for use with the Series 7000 includes QUANT-3, for multicomponent analysis, and the CUV-3 package. CUV-3 contains over 70 commands for acquiring, storing and manipulating spectral data, as well as OBEY programming which allows a series of CUV-3 commands to be strung together to automate specific applications.

*For further information contact Perkin-Elmer Ltd, Post Office Lane, Beaconsfield, Buckinghamshire HP9 1QA, UK. Tel.: 04946 6161.*

**Circle No. 32 on Reader Enquiry Card**

### Hilger's new products

Hilger Analytical's *Newsletter* No. 22 contains information on eight new products together with articles on a clinical laboratory data management system, the spectrochemical analysis of steels, and a data-management package designed to meet the requirements of quality-assurances schemes.

The new products are the Polyvac E980 Series automatic emission spectrometer with Octopus 128K compute and advanced software packages; Chromaspek M amino-acid analyser with microprocessor control, VDU presentation of results and floppy disk storage; Isospek portable, bench-mounted, microprocessor-controlled, radioisotope excited X-ray analyser; Monospek monochromator with holographic grating system, microprocessor-control and stepping motor driver mechanism; Saitron CLP multichannel, computer-controlled discrete analyser; Saitron 9609 microprocessor-controlled single-channel automatic clinical chemical analyser; Saitron 907 microprocessor-controlled photometer with flowthrough system and print-out; and the Saitron 253 electronically controlled automatic diluter.

*More information from Hilger Analytical Ltd, Westwood, Margate, Kent CT9 4JL, UK. Tel.: 0843 25131.*

**Circle No. 33 on Reader Enquiry Card**

### Chromatogram immersion

Visualization of substances after thin-layer chromatography is preferably done by inspection under ultra-violet light. If substances do not respond, or if specific identification reactions are required, suitable reagents have to be applied as uniformly as possible. Liquid reagents can be distributed more evenly by dipping rather than by spraying. The chromatogram has to be immersed and withdrawn at a uniform speed, however, because otherwise 'tide-marks' may be left which could interfere with densitometric evaluation. CAMAG's immersion device effects a vertical movement at a steady speed, and the holding time in the reagent can be pre-selected between 1 and 10 s: in this way, the immersion conditions can be standardized. The device includes two glass dip tanks for HPTLC plates up to 20 × 10 cm in size. A volume of 150 ml reagent is needed to fully immerse the chromatogram (max. 9 cm deep). The dip tanks are designed so that the plate cannot stick fast to the walls.

*Ch. Gfeller at CAMAG, Sonnenmattstrasse 11, CH 4132 Muttenz, Switzerland (tel.: 061 61 34 34), can supply further details.*

**Circle No. 34 on Reader Enquiry Card**

### Pump for media dispensing

The DPS200-RP2 is the new high flow rate version of the Aquarius digital pump system – it has been designed for highly viscous media. The system has a number of accessories to allow the operator to work faster: for example a pneumatic footswitch which leaves both hands free for moving bottles, flasks and tubes under the delivery point. There is total electrical safety in case of spillage. The tubing is held in a flexible arm which is easily moved to a convenient position. Alternatively, a handset is provided for tube-to-tube and petri dish dispensing. Multiple flowlines may be fitted enabling, say, three media to be dispensed in parallel. Once calibrated for the fitted tubing any dose volume may be selected (between 0·1 ml and 999·9 ml with the standard pumphead). Aliquots may be dispensed on demand or in a fully automated cycle when speed, interval and number of steps have been selected. In either mode, Aquarius will display at any time the number of aliquots dispensed so far; a third mode allows Aquarius to pump continuously while updating the display of volume dispensed.

Aquarius is easily interfaced with a variety of other laboratory instruments; typically, a fraction collector whose turntable has moved to the next position can give a pulse to the pump to dispense the pre-set volume of medium. This sytem eliminates the tedium of repeat dispensing. Full computer control is available via a 15-way D connector.

*Aquarius is available in the UK from V. A. Howe & Co. Ltd, 12–14 St Ann's Crescent, London SW18 2LS.*

**Circle No. 35 on Reader Enquiry Card**

### Colour measurement

Some new software programs from Macbeth can speed up production and quality-control measurement of colour whilst reducing operator errors. This 'Quick-Key' software is for Macbeth's 1500 Series Colour Measurement System. It allows the operator to change the parameters of measurements with single keystrokes, avoiding the route through submenus to raise the same information. For instance, the effect of a different illuminant, for example a fluorescent tube, can be obtained by pressing a single function key this saves time and reduces error possibility in the re-setting of the other parameters.

Single key-strokes will access colour difference formulas, measuring 'averaging', pass/fail tolerances, transmission/reflectance modes, illuminants and observer selections. Once a key is dedicated to a function, one key-strokes will bring up a full screen immediately. The Series 1500 System consists of the Macbeth Color-Eye optical sensor linked with a personal computer. Color-Eye works with several computers, including the IBM PC and XT, Olivetti M24, Compaq and DEC Rainbow 100. The latter, for example, has a 104-key layout, for which Macbeth provide a dedicated key overlay or tab strip. A 'Help' key will display on screen the key identification and the next required function.

The system, which works either online, in production, or as a bench instrument in the laboratory, is normally completed with VDU, dual disk drive and the customer's selected hardware and software options. In keeping with the Quick-Key facility, data storage capacity has recently been expanded to allow more than 2000 standards to be stored on one 5·25 in disk.

*Details from Macbeth (UK) Ltd, Bridgewater House, Bridgewater Street, Sale, Cheshire M33 1EQ, UK. Tel: 061 962 6818.*

**Circle No. 36 on Reader Enquiry Card**

### Plans announced for rationalization of BCS and NATLAS into unified laboratory accreditation scheme, NAMAS

The British Calibration Service and the National Testing Laboratory Accreditation Scheme are to be merged into a single unified scheme to be known as the National Measurement Accreditation Service. The aim is to improve efficiency by rationalization of administration. Mr Geoffrey Pattie, Minister of State for Industry and Information Technology, said the merger should be complete by 1 October 1985. Both bodies are operated by the National Physical Laboratory and are based at Teddington. They are essential for laboratory accreditation in the National Measurement System. The terms BCS and NATLAS, which are widely recognized both in this country and abroad, will be retained as groupings within the new Service.

*Background*

The National Physical Laboratory (NPL) is the UK's national standards laboratory and is the focus of the National Measurement System. It provides the national primary standards of measurement together with the calibration services and laboratory accreditation services necessary for their exploitation by industry and others. It also undertakes the research necessary to support these activities and runs a programme of international collaboration to ensure that the UK's measurement system is compatible with those adopted by countries with whom we trade and co-operate scientifically.

Measurement and measurement-related activities in the UK cost an estimated £15 000 million each year.

The BCS accredits calibration laboratories, mainly in the private sector, for competence to carry out specific calibrations for customers. NATLAS has a similar function in connection with testing laboratories.

*More information from the Department of Trade and Industry, 1 Victoria Street, London SW1H 0ET.*

**Circle No. 37 on Reader Enquiry Card**

### MicroGenie

Beckman have announced MicroGenie – a powerful, easy-to-use program designed for the collection, analysis and comparson of nucleic acid and protein sequences.

The program was developed by Dr Cary Queen and Dr Laurence Korn and runs on the IBM PC or XT microcomputer. Operation of MicroGenie is simple with built-in menus and help messages to guide the user at every step.

MicroGenie is available with more than 3000 sequences in 'GenBank' and can search the whole data-bank for sequences compatible to those in the laboratory. A unique sequence editor allows entry and change of sequences quickly, and a sonic digitizer can be used to enter sequences directly from autoradiograms.

MicroGenie determines residue and codon frquencies, translates and reverse translates, locates open reading frames, merges sequences, even predicts RNA and protein secondary structures.

Recent enhancements to the program include Dot Matric Plot, Shotgun Sequencing, Protein Data Base and an update to the GenBank Nucleic Acid data-base.

*Further information, including a demonstration diskette, is available on request from Beckman Ltd, Progress Road, Sands Industrial Estate, High Wycombe, Buckinghamshire, UK. Tel.: Martin Briant 0494 41181.*

**Circle No. 38 on Reader Enquiry Card**

### Compact titrator

The new Mettler DL20 CompactTitrator is a single-method titrator which assures exact automatic repetition of a method, even after a change in operator. The DL20 can be configured for the following titration methods: titrations to a preset endpoint or equivalence point; p and m values (acid and base capacities) in water; TAN/TBN values (total acid or base number according to DIN/ASTM) in petroleum products.

The DL20 is intended for use in plant operations, for example for quality control in production. Provision is made for the interfacing of Mettler balances and printers, sample transports and various commercial printer-plotters. The weighing data are reconciled automatically and recorded. If desired, the DL20 can be operated by a personal computer via an RS232C interface.

*Details from Mettler Instrumente AG, CH 8606 Greifensee, Switzerland. Tel.: 941 22 41.*

**Circle No. 39 on Reader Enquiry Card**

### Sensitive gas flowmeter has increased operating range

Gas flows from less than 0.25 ml/min up to 10 ml/min can be measured with Cook Variometers' gas microflowmeter. This extension of the operating flow range of their standard 0 to 5 ml/min instrument has been achieved with the company's newly introduced flow multiplier. The flow multiplier is plugged in to .he flowmeter's gas connectors and is

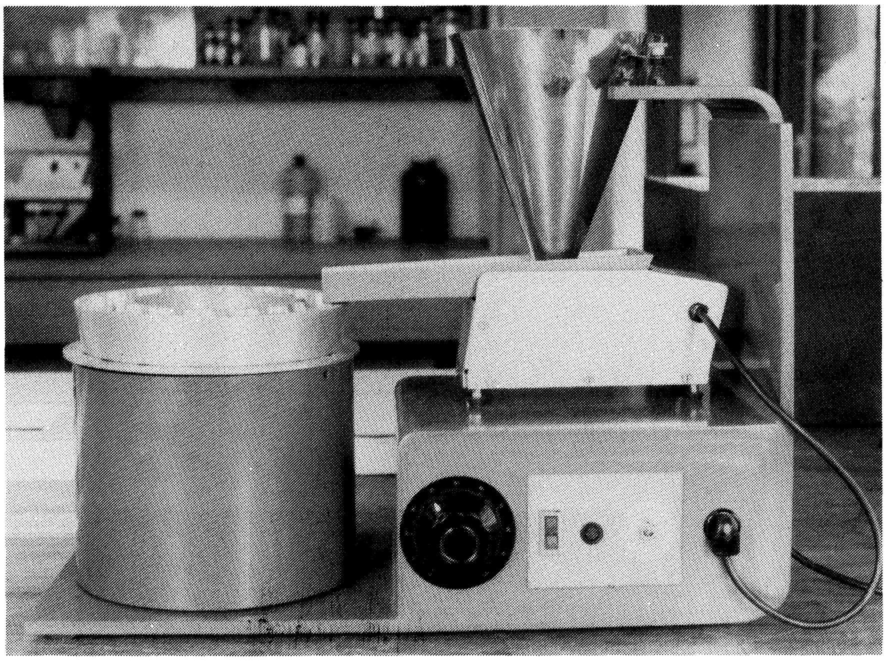

*A low-cost Spinning Riffler for represenative sampling of free-flowing powders, as required for chemical and physical assays relating to industrial processes and developments, from Ladal (Scientific Equipment) Ltd. It features a rotary mechanical dividing head with 16 glass collection tubes for split sampling; and, to generate a uniform media stream overcoming the tendency of powders to separate, incorporates a small vibrating trough feeder. This combines a 1 l hopper adjustable in the height for coarse powder control, and an integral variable controller for 'fine tuning'. Feed rate is adjustable so that each sample is made up of at least 40 increments; this ensures representative sampling. Technical information from Ladal (Scientific Equipment) Ltd, Warlings, Warley Edge, Warley, Halifax, Yorkshire HX2 7RL, UK. Tel.: 0422 56133.* **Circle No. 41 on Reader Enquiry Card**

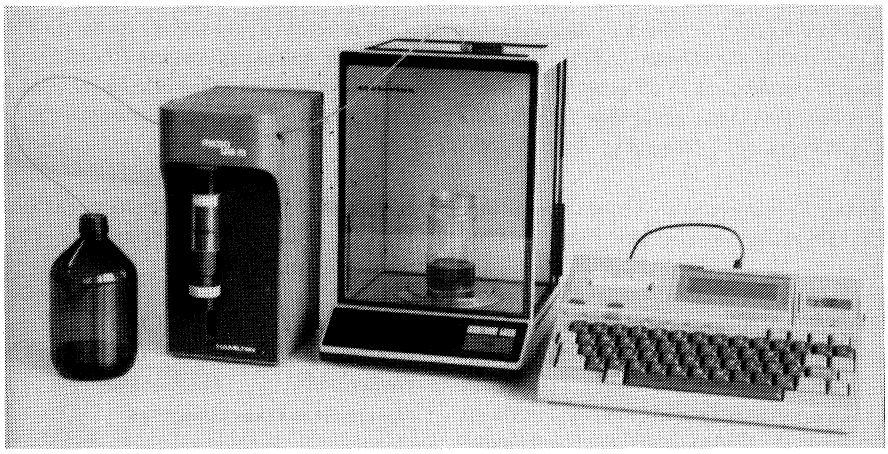

*The preparation of solutions with a certain concentration is a routine operation in every analytical laboratory. Often, the preparation of such solutions is difficult, dependent on skilled operators, and extremely time consuming. Now Hamilton offer a solution: an inexpensive automatic computer-controlled system, combining the MICROLAB M Diluter/Dispenser with a Mettler or Sartorius balance and an EPSON HX-20 computer. This combination allows almost anybody to prepare standard solutions very easily and quickly: the user simply puts an approximate amount of sample on the balance. The computer registers the exact weight of the sample and calculates the amount of liquid required to be added, which is automatically dispensed by the MICROLAB M. In addition, the computer produces a protocol with all the information about the desired concentration in mg/ml, mMol/l, mg% and vol%. Final volumes are available from a few microliters up to 800 ml. The program is based on a dialogue system which leads the user easily through the program. The dialogue is written in English and German on the same microcassette. Further information from Hamilton Bonaduz AG, PO Box 26, CH-74502 Bonaduz, Switzerland. Tel.: 081 37 01 01.* **Circle No. 42 on Reader Enquiry Card**

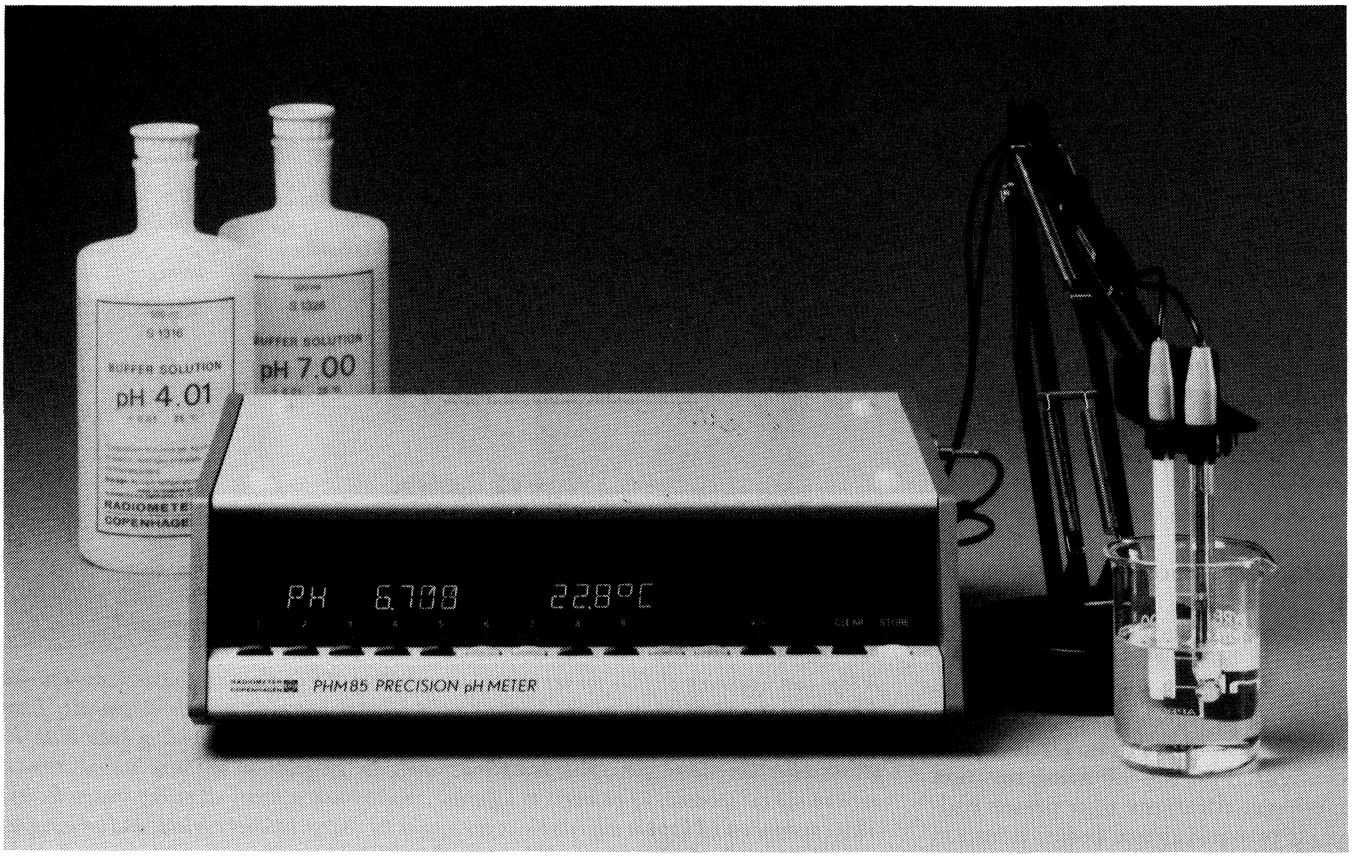

*Radiometer has introduced a pH meter (the PHM 85 Precision ph Meter) for research and high-precision measurement of pH, pX, mV and temperature. The resolution is 0·001 pH, 0·001 pX, 0·1 mV and 0·1 °C. Buffer values can be selected as desired and are easily keyed in and stored in the microprocessor's memory. A 20-character alphanumerical display assists in prompting with clear and concise directions. The operator is kept up-to-date on electrode performance, as the sensitivity, zero-point and ISO-pH point are automatically calculated and displayed during calibration; all calibration data can be recalled for checking by the simple press of a key. PHM85 has RS-232C output for connection to a computer or printer. Routine work can be automated because PHM85 can be connected to a sample changer for automatic measurement of up to 20 samples in series. When connected to an automatic burette, the PHM85 controls automatic end-point titrations. Details from Anne Margrethe Graabaek, Product Manager, Analytical instruments Division, Radiometer A/S, Emdrupvej 72, DK 2400 Copenhagen NV, Denmark. Tel.: 01 696311.*

**Circle No. 43 on Reader Enquiry Card**

fitted or removed in seconds without modification to the standard instrument.

Display is digital in ml/min, and a 0 to 10V output drives recorders, data loggers etc. This fully portable instrument is finding applications in chemical, biological and environmental research laboratories, and as a laboratory standard for calibrating other instruments, capillaries and orifices, and also as a leak detector.

*Details from Cook Variometers Ltd, PO Box 36, High Wycombe, Buckinghamshire HP13 6BB, UK. Tel.: 0494 33171.*

**Circle No. 40 on Reader Enquiry Card**

### 'Products for Chromatography'

A 60-page catalogue with many new products which is available free from J. T. Baker Chemicals. Topics covered include: sample preparation, HPLC, TLC and preparative column chromatography.

*Requests to J. T. Baker Chemicals B.V., PO Box 1, 7400 AA Deventer, The Netherlands.*

**Circle No. 44 on Reader Enquiry Card**

### Modified Autosampler to automate analysis of gels by continuous-flow analysis methods

In conjunction with the Analytical Department of the Kodak Research Division, P. S. Analytical Ltd have developed an Autosampler capable of holding samples at elevated temperatures. Analysis of certain materials can be effectively carried out using standard Autoanalyser procedures providing that the sample, gels at room temperature, are held at 45 °C in a liquid form. Conventional autosamplers are not amenable to modification to retain samples in this state.

P. S. Analytical has modified its Large Volume Autosampler so that the sample tray is held in a water bath held at constant temperature by a feed of water from the recirculating bath. This also feeds water to the analytical system to maintain the elevated temperature during the analysis. Samples held in wide necked bottles sealed with cling-film are then stabilized and then presented to the analytical system under computer control via the probe and wash assembly. The autosampler's simple-to-use TTL interface makes this process easy and reliable. Automated turntable reactivation and the ability to hold the Autosampler in any position pending data from the computer make the system extremely

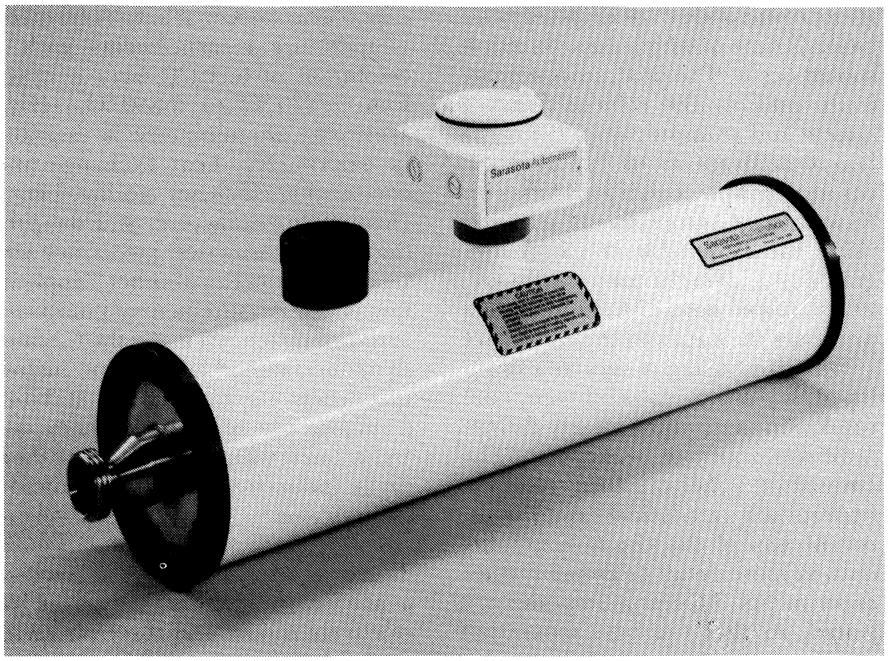

*The new FD830, Hygienic Liquid Density Meter introduced by Sarasota Automation Ltd, the British UK flow and density specialists. Food processing applications include milk, cream, sugar, solution, syrup molasses, egg slurries, beer, lager, soup and fruit juices.*

flexible. Automatic sample repeat under computer control is also an additional feature available on the PSA range. The Autosampler modified as outlined above is PSA part No.20.222 and is available on prompt delivery.

Further models available in addition to the above are the standard 20-position 60 ml sample container version and an 80-position 30 ml sample system.

*Further details on this product and others in the range are avilable from P. S. Analytical Ltd, 2 Eagles Drive, Tatsfield, Westerham, Kent TN16 2PB, UK.*

**Circle No. 45 on Reader Enquiry Card**

### Liquid density meter

A liquid density meter for hygienic applications in the process industries and in pharmaceutical production has been announced by Sarasota Automation Ltd. All wetted parts of the instrument are made from corrosion-resistant stainless-steel with a food-grade nitrile liner fitted to the stainless-steel bellows. A smooth bore, non-obstructed flow-path eliminates the risk of contamination and allows the measurement of almost any product from clean fluids through to calulation of solid content or other density-related values in the most difficult slurries.

Resistant to shock and vibration, the FD830 has a negligible viscosity effect. The meter has a fast dynamic response, measured in ms, and because it has an integral platinum resistance thermometer it will automatically compensate for temperature variations and provide on-line temperature and specific gravity measurement.

The measuring technique of the new device uses a thin-walled tube, which is maintained in transverse oscillation by an electro-magnetic circuit. The frequency of oscillation produces signal relative to the density of the product within the vibrating tube.

The new instrument is available in a stand-alone form or as part of a packaged system incorporating the Sarasota HC900 Density Converter. With this device the system can be arranged to provide a direct output in customer's own units (for example percentage fat, sugar concentration etc.) and relate specific gravity levels to the concentration of a product. Output relays are included to provide high and low limit controls, for example to isolate or initiate pumping on a blending system, and a total of seven output expansion ranges enables selectable control of up to seven different product grades/mixes.

Standard process connections of the unit are 1 in hygienic IDF connectors although SMS, API, DS and DIN standards can also be met. Versions conforming to BASEEFA EX81094 are also available.

*More details from Sarasota Automation Ltd, King's Worthy, Winchester, Hampshire SO23 7QA, UK. Tel.: 0962 883200.*

**Circle No. 46 on Reader Enquiry Card**

### Fourier transform infra-red spectrometer

Perkin-Elmer have announced a new high performance Fourier transform infra-red spectrometer, the Model 1800. The instrument combines the flexibility needed to meet the most demanding research applications with the convenience required for routine operation. Resolution is better than $0.2cm^{-1}$ and the frequency range can extend from the near to the far infra-red.

A double beam optical system overcomes the problems associated with traditional single beam operation. By taking interleaved scans of sample and reference beams, excellent cancellation of atmospheric absorption is achieved without delays for purge stabilization. Careful optical design and the use of full double-sided interferograms ensure extremely good photometric accuracy up to high absorbance values.

The Model 1800 contains, in effect, two interferometers, since two sources, two detectors and two beam-splitters can be installed simultaneously. These can be switched under software control so that a single command can generate a spectrum covering a very wide frequency range. The very high optical throughput leads to excellent signal-to-noise performance, even with room-temperature detectors.

The optical bench of the Model 1800 is pneumatically isolated from external sources of vibration. To avoid disturbance, the interferometer is automatically sealed off when the large sample compartment is opened.

Data acquisition and Fourier transformation are handled by a dedicated processor; the instrument controller is a Perkin-Elmer 7500 Professional Computer, equipped with 640 kilobytes RAM, two floppy disk drives, a Winchester hard disk, and colour CRT. The PE 7500 provides three levels of operation: standard conditions for typical applications, user-generated stored methods, or access to all instrument and data-processing parameters for selection by the operator.

The software provided with the system contains extensive spectral manipulation routines, interactive colour graphics and OBEY programming; QUANT-3 and SEARCH-3 IR applications software are also available, as are such languages as BASIC and FORTRAN 77.

The Model 1800 is designed to accept system expansions. It includes provision for automatic deployment of additional sources, beamsplitters and detectors for research work. Options available in the future will include a GC interface and a variety of sampling accessories.

*For further information contact Perkin-Elmer Ltd, Post Office Lane, Beaconsfield, Buckinghamshire HP9 1QA, UK. Tel.: 04946 6161.*

**Circle No. 47 on Reader Enquiry Card**

### PESOS—solvent optimization software

PESOS has been designed to assist chromatographers in developing liquid chromatography separations for samples containing a known or unknown number of components. The software runs on Perkin-Elmer's 7500 Professional Computer with a Series 4 LC solvent delivery system. It works with any mobile phase system, including normal phase, reverse phase, ion pair and ion exchange. The operator simply instructs the systerm to scout the appropriate variable, i.e. solvent strength, pH, ionic strength, and reviews the results.

PESOS offers an empirical approach to solvent optimization that does not require complex equations that predict retention behaviour. The operator need only select the solvents, search increment and maximum analysis time in which the separation should occur. The program automatically informs the solvent delivery system and autosampler of the number of analyses to be run and the chromatography conditions for each. Raw data are automatically acquired using the Perkin-Elmer Chromatographics 3 data handling software, and stored on hard disk. PESOS then processes the data and the results are summarized using a specially developed, full-colour, multi-dimensional resolution map which clearly defines optimum zones of separation. The chromatographer can then select the appropriate analytical conditions, review any of the chromatographic data in more detail or define further experiments. All chromatograms are stored, so that the best separation can be selected for a particular analysis, whether it be the fastest time, the safest separation (insensitive to minor solvent variations), or the solution that leaves space for additional peaks such as metabolites or internal standards.

Two further features of PESOS are particularly useful. First, since the Series 4 is a four-solvent chromatograph, it is possible to investigate not only a triangular plane that describes a three-solvent space, but also a three-dimensional tetrahedral that describes a four-solvent space. A slice from anywhere in the four-solvent tetrahedral can be cut and displayed, so that the interior of the tetrahedral can be viewed. Secondly, with the colour triangle on the screen, the user can position the cursor over an area of interest and call onto the screen the mobile phase composition and file name for the chromatogram taken at that composition. Chromatographics 3 graphics can then be used to look at the actual chromatogram so that ideal conditions can be selected for the separation.

*For further information contact Perkin-Elmer Ltd, Post Office Lane, Beaconsfield, Buckinghamshire HP9 1QA, UK. Tel.: 04946 6161.*

**Circle No. 48 on Reader Enquiry Card**

### High sensitivity digital thermometer

Automatic Systems laboratories has introduced a digital thermometer capable of absolute and differential temperature measurements with a resolution of 0.001°C over a range from −270 °C to +630 °C. (High sensitivity thermometry is essential in calorimetry, heat exchange and combustion efficiency studies, petrochemical manufacture, and the production of cosmetics, medicines and pharmaceuticals; another application is the measurement of pump and turbine efficiencies using the thermodynamic method.) The new instrument gets its exceptional stability from the use of low frequency AC techniques, which eliminate the thermally induced voltages and drifts in DC systems.

There is a wide range of sensors available, the sensors themselves are relatively robust and this overcomes one of the major limitations of quartz thermometry.

The digital thermometer is microprocessor controlled. Linearization data is held in ROM, and the readout is expressed in °C or °K. RS 232 and IEEE options are available.

*Automatic Systems Laboratories are based at Saxon Street, Linford Wood, Milton Keynes. Buckinghamshire MK14 6LD, UK. Tel.: 0908 320666.*

**Circle No. 49 on Reader Enquiry Card**

### New close-coupled chemical pump features back pull-out facility

A single-stage, close-coupled chemical pump which offers space and weight saving characteristics, is available from SIHI-Ryaland pumps Ltd. Designed to handle clean or turbid liquids in the chemical process and water treatment industries, the pump features a back pull-out facility which keeps maintenance and inspection time to a minimum. The Ryblock RM is suitable for handling liquids up to a temperature of 130 °C; it will operate at 10 bar up to 120 °C and at 8 bar up to 130 °C. The pump is available in sizes ranging from 32 mm to 100 mm delivery branch diameter. Weights range from 56 kg to 287 kg, making it considerably lighter than a long coupled unit. Its close-coupled design allows it to be fitted in very small spaces, as well as avoiding pump and motor alignment problems.

*Full details from SIHI-Ryaland Pumps Ltd, Bridgewater Road, Broadheath, Altrincham, Cheshire WA14 1NB, UK. Tel.: 061 928 6371.*

**Circle No. 50 on Reader Enquiry Card**

### COBAS FARA and COBAS MIRA:

Two clinical chemistry instruments recently introduced in the UK by Roche Products. The COBAS's use computer techniques to provide faster and more precise analyses, simplify data entry and allow unattended operation. Results are presented in the form most convenient to the clinician, as full patient reports, cumulative quality control or statistical breakdowns, with continuous access to patient data.

Centrifugal analysis is probably the most powerful and efficient means of obtaining results from a large number of patient samples simultaneously. The COBAS FARA, utilizing Roche's proven longitudinal light-path system, can perform a wider range of tests than any other comparably-priced centrifugal analyser. Multidirectional, self-aligning pipetting arms automatically select the required sample and reagent, in the exact quantities specified. A fail-safe detection system, which identifies the location of samples and reagents in any of eight interchangeable racks, eliminates the errors inherent in manual pipetting. A pipette-washing cycle guards against contamination, and samples can be prediluted automatically. Samples and/or reagents may be added at any time, and the analytical parameters changed. A simple alphanumeric keyboard and 'talk-you-through' software make operation easy. A 256K memory provides ample storage for programs and results, which are collated or recalculated as required and presented on the screen or in hard copy.

For those laboratories that need to carry out a smaller range of clinical test procedures, the COBAS MIRA has been designed as a compact, low-cost random access system that can also handle batch analyses, all without compromising on accuracy, speed, flexibility or ease-of-use. The COBAS MIRA also has an automated pipetting system which selects the exact quantities of sample and reagent required for each test, and can function as a routine analyser or carry out stat analyses. Washing and sample dilution are automatic, and the system, again operating on the interchangeable rack principle, can handle up to 90 patient samples and select from up to 30 different reagents. Communication is via a simplified keyboard and VDU, and a printer is built in.

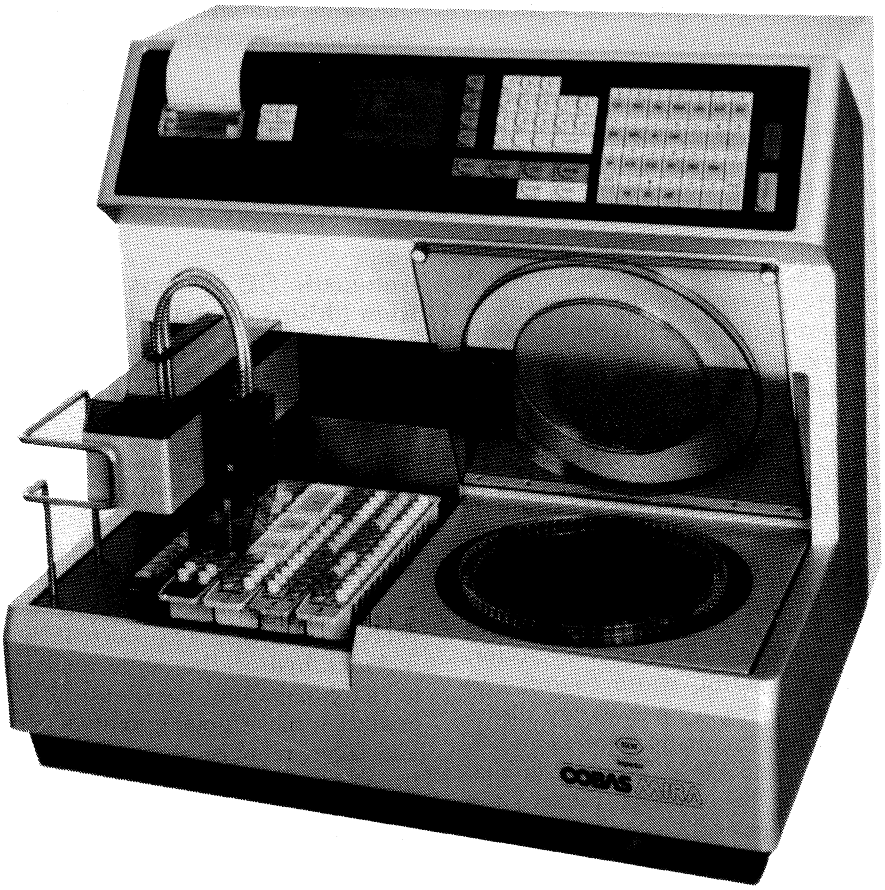

*The new COBAS MIRA, explained by Matt Harris, Roche Products' Marketing Manager, as 'The MIni Random Access analyser—the COBAS MIRA—marks our entry into a market which we feel has not been best served by existing instruments. The MIRA not only beats its rivals in price but utilizes the advanced technology of Roche's centrifugal analysers in such areas as sample handling and data presentation to provide a fully automated, programmable unit that sets a new standard in its field'.*

*Brochures from Jean Kilshaw, Public Relations Manager, Roche Products Ltd, PO Box 8, Welwyn Garden City, Hertfordshire AL7 3AY, UK.*

**Circle No. 51 on Reader Enquiry Card**

### 30-channel peristaltic pump aids automatic analysis

The new Horstmann 30-channel peristaltic pump, available in the UK from MQA (Malcolm Quartly Associates) is designed for precise and reliable operation with almost no maintenance. The unit incorporates an electrical drive/rotor enclosure with a noiseless synchronous motor and a peristaltic pump assembly offering 30 channels for standard colour-coded analytical tubing. Tubing internal diameters range from 0.2 to 1.10 mm with corresponding flow rates from 0.02 to 4.30 ml/min at a pump speed of 5 rev/min. Failure due to tubing wear is eliminated with a patented feature: a PTFE impregnated glass element between the pump rollers and the tubes. Conventional peristaltic pump design of tube location in combed spacers has been advanced by the addition of individual pressure fingers, mounted on a rigid frame, for final and precise tensioning of each tube. Each finger is spring-loaded for efficient operation of large and small bore tubing in adjacent positions giving the smoothest practicable flow pattern and minimum pulsation. The fingers can be removed separately, allowing individual tubes to be replaced while the pump is operating. For fast identification, the fingers are coded and grouped into banks of five.

Standby control is further feature of the peristaltic pump, which enables the operator to vary the duty cycle of the pump motor and facilitate minimal flow when required to avoid wastage of reagents. The synchronous standard motors are virtually noiseless and can be supplied for speeds of 5 or 8 rev/min. Air bar and leak detectors can be fitted to the unit as optional extras.

Industrial applications of the pump include analysis of raw water, process water and effluents in every sector. Quality control, waste control and protein-nitrogen analysis are duties applicable to many process industries including food, brewing, animal foodstuffs, bacteriology, tobacco and research generally. Similarly, applications for these precision pumping units exist throughout clinical analysis and research, and also in the field of chromatography for systems of the Technicon type.

*A descriptive leaflet is available from MQA at 19 Hamilton Drive, Sunningdale, Berkshire SL5 9PP, UK.*

**Circle No. 52 on Reader Enquiry Card**

### RC series printers

A colour technical brochure has been produced by Salter Industrial Measurement Ltd – a subsidiary of Staveley Industries PLC in support of their RC (Range Compatible) series printers. The brochure details the product features of the RC100 ticket printer, RC200 self-adhesive label printer and RC300 UK Department of Trade-approved document printer. A full technical specification of each product is provided together with illustrations of the type of printout which each printer can produce.

Particular emphasis is given to the system and interface capabilities of the printers as system peripherals. Each printer can be linked to counting scales, bench scales, or weighing platforms with weightmeters to provide complete weighing systems.

*Copies from Salter Industrial Measurement Ltd, George Street, West Bromwich, West Midlands B70 6AD, UK. Tel.: 021 553 1855.*

**Circle No. 53 on Reader Enquiry Card**

### Automatic GC systems brochure from Philips Analytical

The latest range of automatic gas chromatography systems from Philips Analytical is detailed ion a new brochure. Based on gas chromatographs, integrators and accessories from the Pye Unicam range, the systems are factory-engineered by the company's Projects Group for a wide variety of standard industrial process analyses. The current list includes hydrocarbon-type analysis of naphthas and gasolines (PNA analysis), the original gravity of beer and lager, refinery gas, natural gas, transformer oil gas, sulphur gas, simulated distillation, nuclear power generation, ammonia and LPG.

An applications service for customers requiring factory-engineered GC systems for their own particular automatic or on-line analyses is also provided by the Projects Group, together with a design, installation and testing service of complete column packages for specific tasks.

*The brochure can be obtained from Pye Unicam Ltd, York Street, Cambridge CB1 2PX. Tel.: 0223 358866.*

**Circle No. 54 on Reader Enquiry Card**

### Microprocessor RGA with built-in data storage

VG Gas Analysis decribe their Micromass Residual Gas Analyser as amongst the most versatile in its price performance area. A wide screen monitor permits display of the extensive software package including analogue and histogram displays, split screen library, peak selection with high and low alarm outputs and three decade trend analysis. A multi-track tape data storage system allows results to be stored on board and reviewed later; alternatively, RS232 or IEEE interfaces are available for host computer control. A high-pressure operation capability (to $10^{-3}$ Torr) of the Faraday Analyser makes it ideal for applications which are borderline for other RGAs. A dual detector version is available for those needing the lower detection limits and faster scan speeds of an electron multiplier. The mass range of the unit is 1–100 AMU and it is expected to find application in many vacuum processes including splluttering, evaporation, crystal growth, reactive ion and plasma etching and tube manufacture.

*Further information is available from VG Gas Analysis (Quadrupoles Division) Ltd, Nat Lane, Winsford, Cheshire CW7 3QH, UK. Tel.: 06065 50111.*

**Circle No. 55 on Reader Enquiry Card**

### ICP Methods Manual from Allied Analytical Systems

Allied Analytical Systems offers a *Methods Manual for Inductively Coupled Plasma Emission Spectrometry,* a 230-page reference book to help analysts reduce the time and guesswork normally involved in developing and verifying analytical programs. The manual comes in a three-ring binder format to allow easy updating and insertion of new technical information.

The manual provides the analyst with a comprehensive discussion of actual emission spectra recorded on the ICP instrument. It includes recommendations applicable to the normal nebulization of aqueous solutions, and it covers the measurement of emission lines in the conventional UV-visible region of the spectrum (190–900 nm).

The stored information of the manual is complete with guidance for choosing wavelengths for up to 33 different elements. Video screen reproductions depict baseline structure and spectral interference at 2–6 wavelengths per element. Also, the information will help an analyst quickly write an analytical procedure for a new sample type.

Section One of the manual covers system variables, program variables, and element variables. Section Two includes a checklist for ensuring proper ICP performance and for verifying analytical methods. Section

Three discusses sample preparation and emission wavelengths and covers the most frequently used emission lines for the elements most often determined in water and wastewater.

*For more information contact Allied Analytical Systems, One Burtt Road, Andover, Massachusetts 01810, USA.*

**Circle No. 56 on Reader Enquiry Card**

### Tube from Draeger Safety detects formaldehyde

A Formaldehyde detector tube is the latest addition to the Draeger Safety range of over 200 tubes which signal the presence of gases and toxic vapours in an atmosphere. This tube is specifically designed to detect Formaldehyde at concentrations as low as 0.04 ppm. By providing measurement below the World Health Organization recommended safety threshold limit, the tube gives adequate prior warning of a potentially hazardous situation. (Formaldehyde can be found during the manufacture of chip-board articles such as furniture, insulation material such as cavity wall foam, textiles, carpets and paints. Relatively low concentrations can cause irritation to the eyes and skin and Formaldehyde is a suspected carcinogenic.)

*Details from Draeger at Sunnyside Road, Chesham, Buckinghamshire HP5 2AR, UK. Tel.: 0494 774481.*

**Circle No. 57 on Reader Enquiry Card**

Draeger Safety also offer a colour leaflet which describes the benefits, features and applications of the Chloralarm system, a chlorine and bromine monitor.

**Circle No. 58 on Reader Enquiry Card**

### 'Innovation in analysis'

Phillips Analytical launched a number of new products to the scientific press during the Autumn of 1984. The instruments included spectrometers, microscopes, chromatographs, and a pH and conductivity meter; of course some of these 1984 introductions have already been covered in *JAC*. We report on the PW 1404 and 1606, the SEMs and Edax 9900 and the PU 4900 here.

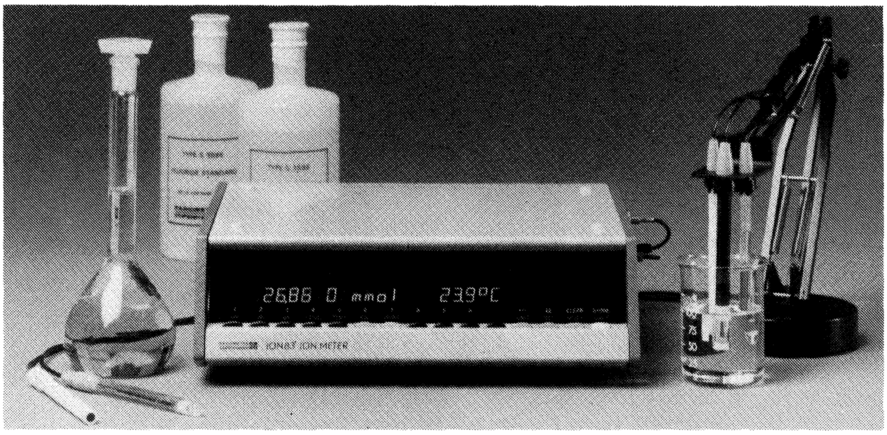

*Radiometer's ION83 ion meter for precision measurement and routine work with pH- and ion-selective electrodes. Ion concentration is determined by direct potentiometry. Multipoint calibration with up to five standards including a blank assures accuracy and a full working range of electrodes. Measurements with several electrode pairs is simplified, as the ION83 has the capacity for storing the calibration data for up to three different electrode pairs individually in its memory. Routine measurements can be completely automated with the ION83, as it can be directly connected to a sample changer, handling up to 20 samples in series. A 20-character alphanumeric display prompts the operator and displays the results in appropriate units. RS-232-C output allows the ION83 to be linked to a printer or a computer. The UK distributor is V. A. Howe & Co. Ltd, 12–14 St. Ann's Crescent, London SW18 2LS.* **Circle No. 59 on Reader Enquiry Card**

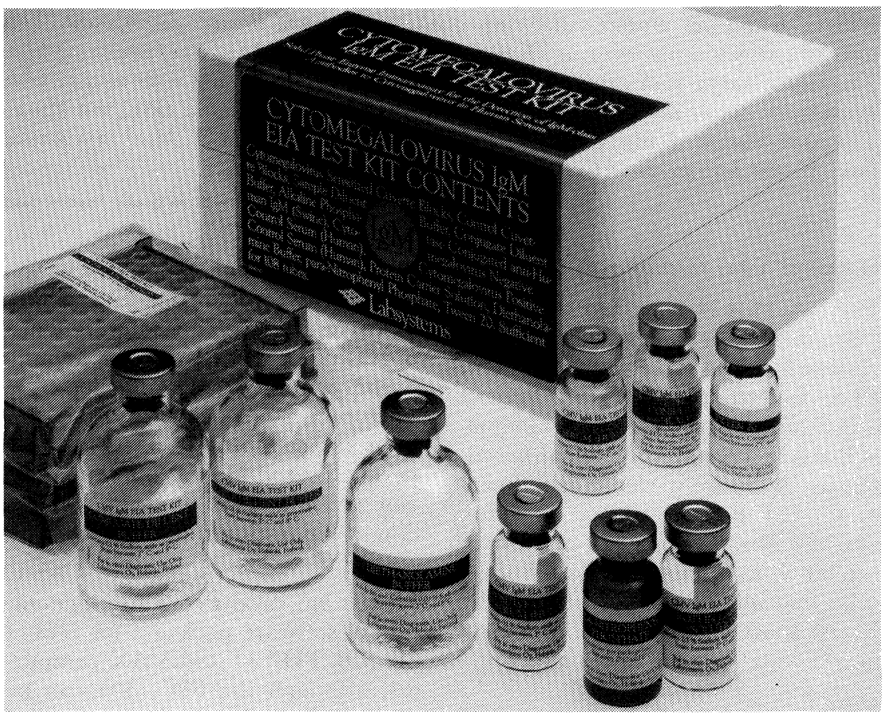

*With the addition of a further Cytomegalovirus (CMV) test kit, Labsystems now offers kits for the accurate detection of both IgM and IgG class antibodies to CMV infections. The new IgM and the IgG tests are based on solid-phase enzyme immunoassay, automatic photometric reading and simple laboratory work. They are safe, easy to use and require only 4 h to produce results. The assays speed up CMV diagnosis considerably, especially in large screening tests. A positive IgG test suggests either previous or acute infection. A positive CMV IgM test suggests acute and recent infection. An accurate evaluation can be made with only one serum sample. These tests represent a reliable method for immunity surveys; diagnosis of congenital infections; diagnosis of acute CMV infections and testing the quality of fresh whole blood to be transfused to transplant and immuno-suppressed patients. All relevant reagents are contained in the test kits, together with CMV antigen sensitized cuvette blocks and control cuvette blocks. Further information from Labsystems (UK) Ltd, 12 Redford Way, Uxbridge, Middlesex UB8 1SZ UK. Tel.: 0895 38421.*

**Circle No. 60 on Reader Enquiry Card**

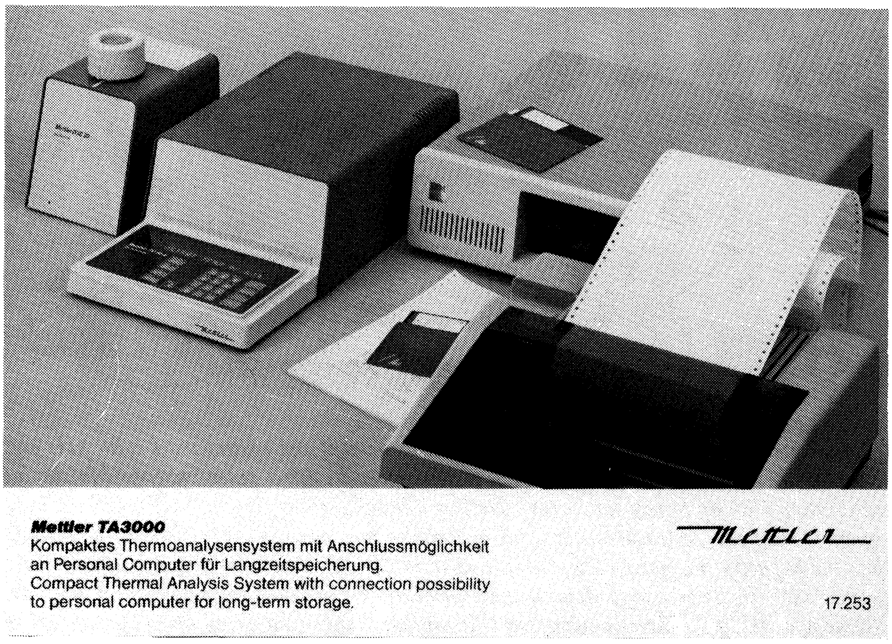

*A number of features have been added to Mettler's TA3000: a data interface for connection of a personal computer, a new measuring sensor for Differential Scanning Calorimetry (DSC), and adaptation of the comprehensive evaluation programs to the latest needs of the user. The TA3000 can be used in any location requiring qualitative and quantitative material testing for product monitoring, quality control and product development. This compact system is composed of the TC10A control instrument and a series of measuring cells. This means that for each application – be it routine analysis or research – the most suitable combination can be configured for each task. This not only applies to DSC, but also to Thermomechanical Analysis (TMA) and Thermogravimetry (TG). More information from Mettler Instrumente AG, CH-8606 Greifensee, Switzerland.*

**Circle No. 61 on Reader Enquiry Card**

*Sequential X-ray spectrometer*

The PW 1404 is described as having a number of significant advances in hardware and software design. Developed from the PW 1400 Series, it can now be provided with unique multilayer 'crystals' giving enhanced sensitivity to light elements – down to C and B. The choice of side-window X-ray tubes has also been extended to include a dual-anode tube that allows the use of a single X-ray source without compromises over a wide range of applications.

A compact, high-efficiency generator is built into the spectrometer to give a space-saving one-cabinet system, while providing 100 kV programmable capability that is particularly valuable for heavy element determinations. Other notable features are improved detector design, an auxiliary high resolution collimator and programmable channel masks that reduce background.

High speed measuring electronics and fast digital scanning allow rapid data collection for qualitative analysis, and a compact loading airlock increases sample throughputs when operating under vacuum or helium. System operation is controlled by a microprocessor – which also contains sufficient analytical software to permit stand-alone operation in emergencies, plus a range of self-diagnostic testing routines.

Philips has produced a new generation of software packages for DEC's PC 350, PDP 11 and VAX computers, although the PW 1404 can be connected to any other processor preferred by the user. The standard X44 software provides sophisticated qualitative and quantitative capabilities, including the functional use of colour graphics. It nevertheless remains extremely easy to use, with simple menu and dialogue presentations, extensive help messages and built-in error protection. As a further aid, the software is available in French, German and Spanish, as well as English. Quantitative programs can be assembled using a choice of five calibration models, including Claisse-Quintin and a new Philips model that provides high accuracy over wide measuring ranges. For qualitative analysis, specially modified Garbauskas and Goehner programs are optionally available, giving broad scope for spectral manipulation and display.

Output possibilities include transmission to remote terminals or computers, with flexible report formatting and editing to suit user requirements.

A range of sample loading systems is available.

*Simultaneous X-ray spectrometer*

The PW 1606 is a powerful system for process-control analysis in such industries as cement, steel, non-ferrous metals, glass and mining. The proven optical design of the PW 1600 Series has been retained, permitting high-speed measurement of up to 28 elements. One or two of the fixed channels may alternatively be replaced by compact programmable goniometers, to give increased versatility for qualitative analysis and the determination of non-routine elements. With compact solid-state electronics and a high-efficiency generator small enough to fit inside the spectrometer itself, the PW 1600 is now produced as a one-cabinet instrument. A 3 kW, 60 kV generator cuts power demand by up to 40%, and eliminates the need for a special cooling circuit. Operating economy is further improved by an extended range of detectors, which enable all elements above Mg to be measured without external gas supplies. Spectrometer functions are controlled and supervised by microprocessor, and the provision of a universal interface allows data reduction to be carried out using any computer selected by the user. The latter feature is intended to make the PW 1606 an ideal component of automated laboratories and centralized plant control systems.

*Extra-capacity chamber and large stage movement for SEM 525*

A specimen chamber measuring 310 mm high by 380 mm wide and 280 mm deep with a stage that moves 100 mm in both *x* and *y* directions are major features of the SEM 525 from Philips's Electron Optics Group. The SEM 525 is one of the Series 500 family of microscopes. It was designed for ease-of-use in routine applications, but is equally suitable for advanced research. The large specimen capacity will be of particular interest to the semiconductor industry, general materials and metallurgical laboratories. Improved gun geometry gives high resolution and a better signal-to-noise ratio. In a special low Kv configuration this provides a five-fold increase in brightness when compared with the standard configuration.

*Microanalytical system*

Edax 9900, a new product from Edax International, Inc., Prairie View, Illinois, has been introduced by Philips. The system can collect, process, display, and store a host of signals from electron microscopes, either SEM or STEM. These signals include: backscattered electron (BS), secondary electron (SE), absorbed electron current(AE), cathodluminescence (CL), EDS elemental X-ray, WDS elemental X-ray and EELS (Electron Energy Loss Spectrometry). A full complement of image presentation software and the Edax Digital Scan Generator – the EDSG – are part of the 9900 imaging system and are used to collect and process this data. The imaging presentation software allows collection matrices up to 1024 × 800 points. The specific dwell time and the area size of the scan are completely user programmable.

Quick viewing of the collected image can be obtained through a rapid scan and display mode. Available processing functions include signal thresholding, signal ratioing, background subtraction and deadtime correction. Storing of images is done easily and routinely in a number of ways: points as they are being collected (real time), processed image after various enhancements, and displayed image. Various histogram comparisons assist the user in assessing current images and manipulating data.

A palette of 256 colours from a total of over 16 million colours allows the user unlimited area of graphics presentation. Stored images can be

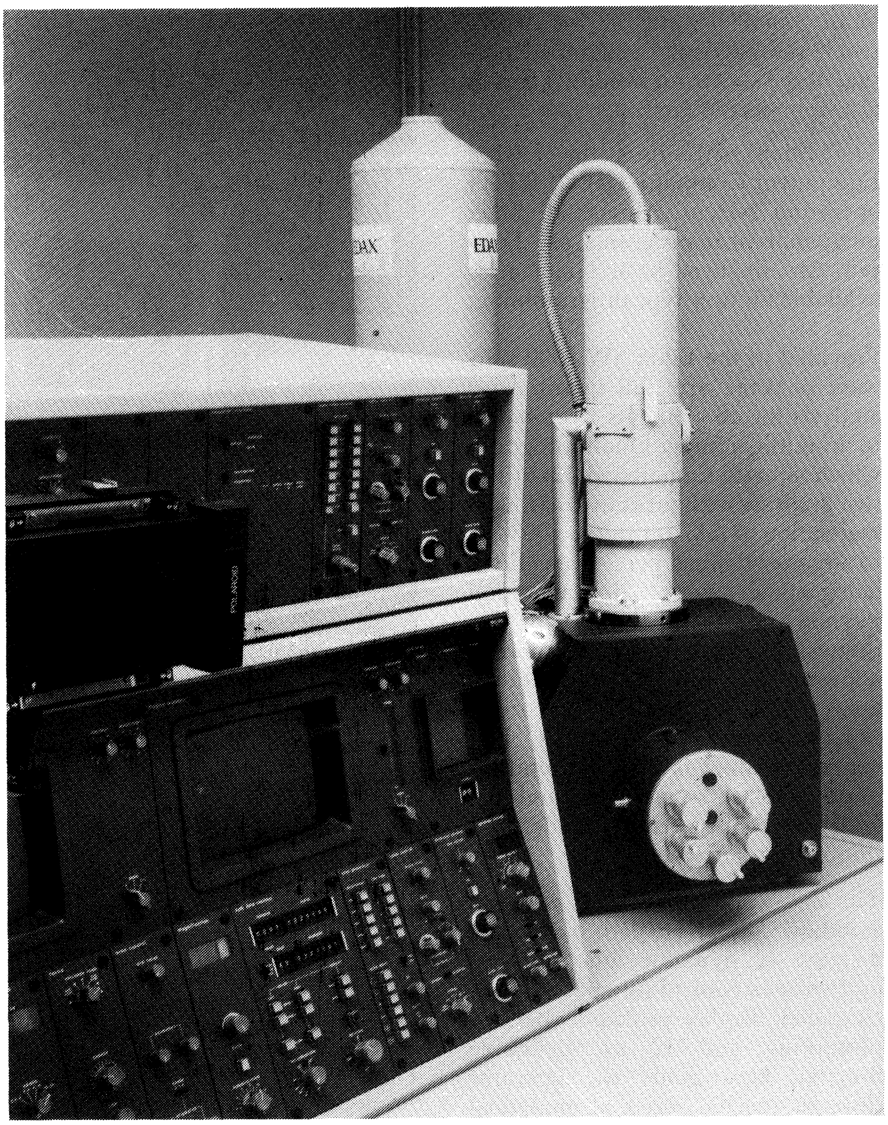

*An entirely new feature of the SEM 525 is the opportunity to include microprocessor-controlled automatic focussing and astigmatism correction. Based on algorithms developed by the Philips research laboratories in Hamburg, fully automatic focussing and astigmatism correction is achieved – even in the most difficult circumstances.*

manipulated mathematically for composite imaging such as addition, subtraction, ratioing and signal mixing. There are also mathematical models for smoothing with neighbourhood averaging (low pass filter), edge sharpening via discrete differentiation (high pass filter), and image restoration versus gradient interpolation.

The Edax digital scan generator provides full communication with and automation of the electron microscope. It generates remotely a computer controlled scan with a specific array size and dwell time, then rapidly digitizes a specific EM signal, and stores and displays this information within the 9900.

An ultra-high resolution colour display system is standard with the 9900. Since it is RGB compatible, it has a pixel (picture element) resolution of 512 horizontal × 400 vertical. Each pixel is individually addressable.

A fully rational display mode is available for EDS. Standard features are elemental identification symbols, spectral labelling, horizontal energy range and the Edax magnifying glass – a new approach to highlighting a field of interest, which gives the user an indication, prior to spectrum expansion, of the next field of view. It eliminates the need for spectrum slewing and provides a means for the operator to demonstrate where, within the total spectrum, the particular magnified portion of the spectrum resides. There is also split-screen facility.

The Edax 9900 uses the Digital Equipment Company (DEC) RT-11 operating system. Almost all Edax microanalytical programs use Fortran. An advanced basic language is available for user type programming.

Provided in the Edax SW 9900 software package are: full quantitative analysis for all elements from boron to uranium, a Super Quant program, which allows unmatched quantitative analysis without standards, normalization of ZAF output to 100% or to any user-defined percentage, Edax halographic peak deconvolution method – a novel technique for distinguishing peak overlaps, thereby allowing improved quantitative analysis, an advanced ZAF correction program, user-definable PEI factors, Kramer law background fit, multiple least squares fit, and the Hall and Cliff-Lorimer models for thin section analysis.

The 9900's operator console has a full standard ASCII keypad, a message display, 12 dynamic function keys and various control functions. A 96-character display provides operator prompting and 12 user-definable function keys guide the operator through specific series of analytical routines. Each key is positioned near a four-character prompt, used to define the analytical choices available at that level of the program – in essence, a built-in operator's manual. A help key brings up a display of current operation status and further operation guidance.

*Total 'system-power'*

Instead of adopting an uncoordinated add-on data system, the PU 4900 GC's design overcomes traditional limitations by using a single program for both manipulation of data and control of the analytical separation. It also provides a practical platform from which to explore the enormous potential of increasingly popular multidimensional techniques. PU 4900's total 'system-power' comprises a number of special features. The instrument has been optimized for capillary columns with new capillary injectors, a newly-designed column oven and a new range of detectors providing improved separating power, increased sensitivity and reduced analysis time. Although the PU 4900 can be used without limitation for any gas chromatography method or application, it is unique in offering automatic fast capillary analysis.

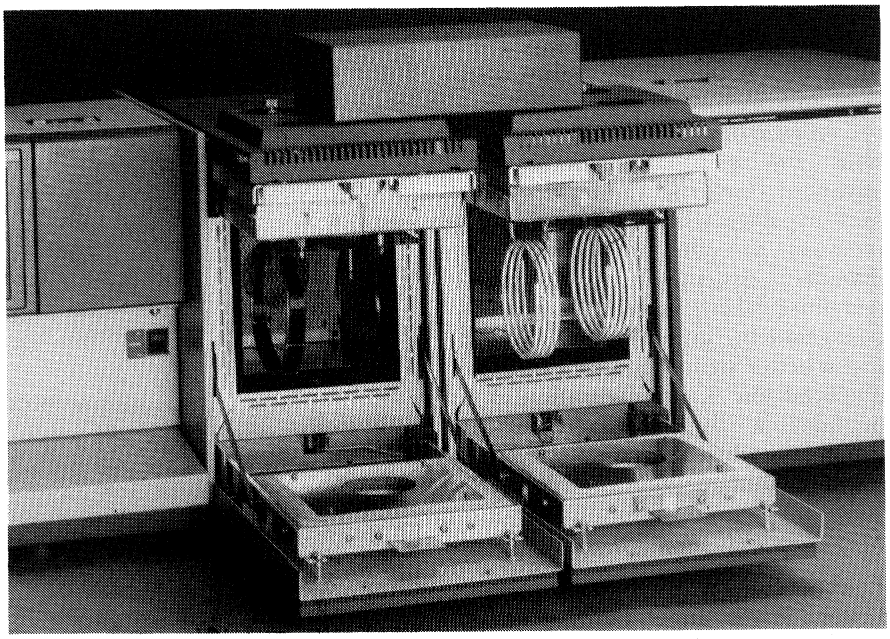

*The PU 4900's satellite oven, although of the same design as the main oven with no compromise in performance characteristics, is a mirror image of the main oven. This means that the satellite oven can be stood side by side for direct transfer of sample from one oven to the other in a multi-dimensional system or can function as a second, independent analysis channel.*

This has been made possible by the development of a new column oven, in which an aerodynamic air flow path ensures that the column is mounted in a thermally stable zone with minimum gradients and high control. The result is repeatability without distortion or splitting of peak shapes. The new design also means that there is an exceptionally low thermal lever (LTL), whereby the direct effect of ambient temperature on the temperature inside the oven is minimized.

The PU 4700 auto-injector gives the basis for total system automation from injection to calculation of results, and it is aided by the PU 4900's fast processing power, storage of up to 12 methods in non-volatile memory, a graphics capability via the integral visual display unit and data storage using twin floppy disks.

All PU 4900 detectors – flame ionization, nitrogen, thermal conductivity, electron capture and flame photometric – have been designed to provide accurate peak signal reproduction. Although they are primarily intended for operation with capillary columns, a unique interface is provided to allow packed columns to be used if required.

A 'mirror image' satellite oven design means that the systemn is an important development in multidimensional chromatography. The satellite oven is identical to the instrument's main oven: the two can stand side by side for direct transfer of sample from one to the other. Ovens are controlled independently but simultaneously and, if MDC is not required, the satellite can be used as a second, independent channel of separating power. The added advantages of comprehensive gas control, the possibility of mounting a sampling and switching valve oven, plus the capability of the PU 4900, with its satellite oven, to accept up to three detector systems operating simultaneously with full data handling facilities for each channel, make the system the first-ever non-dedicated, practical platform for exploring and exploiting the MDC technique.

Whether it is being used conventionally, as a capillary analyser or for multidimensional techniques, the PU 4900 is capable of producing analytical results of consistently high quality. Sophisticated data handling routines are combined with a menu-driven user interface to provide both power and ease of use for the working chromatographer. Reliable self-diagnostics and comprehensive monitoring ensure complete analytical integrity.

*Information from Philips Electronic Instruments, Inc., 85 Mokee Drive, Mahwah, New Jersey, 07430, tel.: 201 529 3800; Philips Export B.V., Scientific & Industrial Equipment Division, PO Box 218, 5600 MD Eindhoven, The Netherlands, tel.: 31 40 782285; or Pye Unicam Ltd, York Street, Cambridge, UK, tel.: 0223 358866.*

**Circle No. 62 on Reader Enquiry Card**

### Automatic p50 curve plotter

The Model B Hemox-Analyzer is an automatic system for plotting the blood oxygen association or dissociation curves of normal, abnormal and cord blood. Only one drop of blood and 10 min recording time are required to obtain a complete curve, from which the p50 value is easily obtained.

Features of this blood oxygen equilibrium analyser include a built-in pressure vacuum pump for sample oxygenation and sample removal after plotting of the curve, as well as flushing of the sample cuvette. A Teflon valve-system provides for easy switch-over of gases and assures corrosion-free liquid delivery to the sample cuvette. The built-in electronic heater system allows for operation at 37 °C without the need of a water-bath.

The front-mounted control panel features a digital-meter for monitoring of temperature, oxygen partial pressure and signal level outputs.

*More information from TCS-Medical Products Co., 2793 Philmont Avenue, Huntington Valley, Pennsylvania 19006, USA.*

**Circle No. 63 on Reader Enquiry Card**

### Anti-HTLV 3 assay

The Du Pont Company has begun clinical evaluations in Europe of an assay to detect antibodies to a virus believed associated with the Acquired Immune Deficiency Syndrome (AIDS). The test will allow blood-banks and hospitals to screen donated blood to help prevent the spread of transfusion-acquired AIDS.

If the clinical evaluations conducted at various European test locations are successful, the test could be commercially available to blood-banks and hospitals in 1985. In this assay, antigens from disrupted human T-Cell Lymphotropic Virus Type 3 (HTLV-3) are coated on the wells of a microplate. Typical ELISA procedures are then followed to detect the antibodies which may be present in donor serum or plasma.

HTLV-3 was isolated at the US National Cancer Institute under a research programme designed to study AIDS and Du Pont and Biotech Research Laboratories, Inc., USA, are jointly licensed by the US Department of Commerce to develop and manufacture the test in conjunction with protocols established by the US Department of Health and Human Services. It is estimated that more than 20 million tests will be required annually in Europe alone.

*Details from G. R. A. Lambert, Du Pont de Nemours International S.A., PO Box, CH 1211 Geneva 24, Switzerland.*

**Circle No. 64 on Reader Enquiry Card**

### 'UK Robotics Research'

A new book from Mechanical Engineering Publications Ltd which collects papers read at an Institution of Mechanical Engineers conference held in December 1984. Topics discussed, by speakers from industry and universities, included the evaluation and philosophy of robot installations, sensors, drives, simulation techniques, and applications. The books cost £18.00 (UK) and £23.50 (Overseas).

*Mechanical Engineering Publications are based at PO Box 24, Northgate Avenue, Bury St Edmunds, Suffolk IP32 6BW, UK.*

**Circle No. 65 on Reader Enquiry Card**

### 'Capillary Column Chromatography'

A new 16-page brochure on capillary column gas chromatography is available free of charge from Perkin-Elmer. The brochure describes in detail the various injection techniques for capillary chromatography, gives information on selecting columns, column mounting and connections, and includes advice on achieving automation and precision of analysis with capillary columns through the use of an autosampler.

*For a free copy apply to Perkin-Elmer Ltd, Post Office Lane, Beaconsfield, Bucks HP9 1QA, UK. Tel: 04946 6161.*

**Circle No. 66 on Reader Enquiry Card**

### Carcinogens in drinking-water

A new GC methodology by K. Grob and A. Habich claims to allow the detection of halocarbons in potable water in the fractional/ppb range by direct aqueous injection and the use of an electron capture detector without the problems previously associated with this technique. This development follows studies showing that chlorine reacts with water to produce chloroform – amongst other substances – and the growing industrial use of halogenated hydrocarbons in industrial solvents, with the increased possibility of water contamination. Existing procedures for determining these substances are time consuming, difficult to calibrate and very different to automate.

Amongst basic requirements are the use of a high temperature (350°C) ECD system operating in constant frequency mode. The required column is 30 m × 0.32 mm ID, PS 255 (5 μm film thickness) operating at 104°C (isothermal). An important aspect is the use of a cold on-column injector with secondary cooling to prevent sample back-flow.

Carlo Erba is the only manufacturer offering a system – the AS 550 – able to automate the cold on-column injector. Normally able to handle 42 samples for sequential analysis, the AS 550 has been designed to operate in conjunction with the HEC 960 laboratory computer, allowing a whole sequence of analyses to be run from a floppy disk.

*Full technical information from Erba Science (UK) Ltd, Headlands Trading Estate, Swindon, Wiltshire SN2 6JQ, UK. Tel.: 0793 33551.*

**Circle No. 67 on Reader Enquiry Card**

